# Effects of Running on Sand vs. Stable Ground on Kinetics and Muscle Activities in Individuals With Over-Pronated Feet

**DOI:** 10.3389/fphys.2021.822024

**Published:** 2022-01-13

**Authors:** AmirAli Jafarnezhadgero, Nasrin Amirzadeh, Amir Fatollahi, Marefat Siahkouhian, Anderson S. Oliveira, Urs Granacher

**Affiliations:** ^1^Department of Sport Managements and Biomechanics, Faculty of Educational Sciences and Psychology, University of Mohaghegh Ardabili, Ardabil, Iran; ^2^Department of Sport Physiology, Faculty of Educational Sciences and Psychology, University of Mohaghegh Ardabili, Ardabil, Iran; ^3^Department of Materials and Production, Aalborg University, Aalborg, Denmark; ^4^Division of Training and Movement Sciences, Research Focus Cognition Sciences, University of Potsdam, Potsdam, Germany

**Keywords:** flat feet, loading rate, lower limb mechanics, unstable walkway, muscle

## Abstract

**Background:** In terms of physiological and biomechanical characteristics, over-pronation of the feet has been associated with distinct muscle recruitment patterns and ground reaction forces during running.

**Objective:** The aim of this study was to evaluate the effects of running on sand vs. stable ground on ground-reaction-forces (GRFs) and electromyographic (EMG) activity of lower limb muscles in individuals with over-pronated feet (OPF) compared with healthy controls.

**Methods:** Thirty-three OPF individuals and 33 controls ran at preferred speed and in randomized-order over level-ground and sand. A force-plate was embedded in an 18-m runway to collect GRFs. Muscle activities were recorded using an EMG-system. Data were adjusted for surface-related differences in running speed.

**Results:** Running on sand resulted in lower speed compared with stable ground running (*p* < 0.001; *d* = 0.83). Results demonstrated that running on sand produced higher tibialis anterior activity (*p* = 0.024; *d* = 0.28). Also, findings indicated larger loading rates (*p* = 0.004; *d* = 0.72) and greater vastus medialis (*p* < 0.001; *d* = 0.89) and rectus femoris (*p* = 0.001; *d* = 0.61) activities in OPF individuals. Controls but not OPF showed significantly lower gluteus-medius activity (*p* = 0.022; *d* = 0.63) when running on sand.

**Conclusion:** Running on sand resulted in lower running speed and higher tibialis anterior activity during the loading phase. This may indicate alterations in neuromuscular demands in the distal part of the lower limbs when running on sand. In OPF individuals, higher loading rates together with greater quadriceps activity may constitute a proximal compensatory mechanism for distal surface instability.

## Introduction

There is compelling evidence that the regular performance of running exercise has positive effects on both, markers of health and physical fitness (Thompson et al., 2003). However, a negative side effect of running exercise is the high rate of musculoskeletal injuries. On average, 100 h of running exercise results in one running-related injury (Requa et al., 1993). A prominent running-related injury risk factor is over-pronation of the feet (OPF) (Aryana and Artha, 2019). OPF prevalence rates range between 2 and 23% in adults (Dunn et al., 2004). There is evidence that OPF during the stance phase of running might result in a hyper-flexible, and thus unstable foot (Cheung and Ng, 2007). Moreover, it may create excessive movement transferred into tibial rotation (Hintermann and Nigg, 1998).

In terms of physiological characteristics, OPF has been associated with distinct muscle recruitment patterns (Murley et al., 2009). OPF may develop from deficits in muscle strength, joint instability, and/or overstrain due to the long-term usage of lower limbs structures (Ashnagar et al., 2019). It has been proposed that the triceps surae, peronei, tibialis anterior/posterior muscles act as dynamic stabilizers of the medial longitudinal arch (Thordarson et al., 1995; O’Connor and Hamill, 2004). Accordingly, the foot muscles appear to play a vital role in stabilizing the medial longitudinal arch (Thordarson et al., 1995; O’Connor and Hamill, 2004).

Surprisingly little is known on the effects of exercise programs on muscle activities and function in OPF individuals. A previous study demonstrated that an 8-weeks exercise program designed to strengthen the intrinsic foot muscles improved the medial longitudinal arch in adult patients with chronic ankle instability and OPF (Chung et al., 2016).

Running on sand could be a promising exercise intervention for OPF treatment because it is rather easy-to-access and produces hardly any costs. From a health-related perspective, the unstable element sand may strengthen foot and ankle muscles, improve function, and reduce OPF (Pinnington et al., 2005). Due to these positive characteristics, recreational and high-performance athletes and coaches have previously used running on sand as a successful adjunct to firm surface training regimes (Wischnia, 1982). The increased energy cost of running on sand compared with firm surface running can partly be attributed to an increased activation of distal lower limb muscles associated with greater hip and knee range-of-motion (Pinnington et al., 2005). Of note, sand is a yielding surface that provides constant instability during running. There is evidence from previous studies showing that running and walking on sand compared with stable ground has a major impact on kinematics and kinetics in healthy individuals and patients with multiple sclerosis (Pinnington et al., 2005; van den Berg et al., 2017). A previous study demonstrated lower gait speed when walking on sand compared with stable ground in OPF individuals. In addition, lower peak positive free moments were found during the push-off phase and lower loading rates during the loading phase (Jafarnezhadgero A. et al., 2019). A comparison between walking and running at comparable speeds has demonstrated a number of specific differences concerning ground reaction forces (GRFs; e.g., peak GRFs amplitudes in three dimensions) and muscle activity (e.g., gastrocnemius muscle) patterns (Grillner et al., 1979; Thorstensson et al., 1982; Lichtwark and Wilson, 2008). Accordingly, it is timely to examine this research question during running in OPF individuals. To the authors’ knowledge, there is no study available that examined the effects of running on sand vs. stable ground in OPF individuals. Therefore, the aim of this study was to contrast the effects of running on sand vs. stable ground on GRFs and activities of selected lower limb muscles in OPF individuals compared with healthy age-matched controls. With reference to the relevant literature (Pinnington et al., 2005; van den Berg et al., 2017), we hypothesized lower loading rates, and higher muscle activities when running on sand compared with stable ground in both experimental groups. We further expected that these surface-related effects are more pronounced in OPF individuals compared to the healthy controls (Murley et al., 2009).

## Materials and Methods

### Experimental Design

**FIGURE 1:**
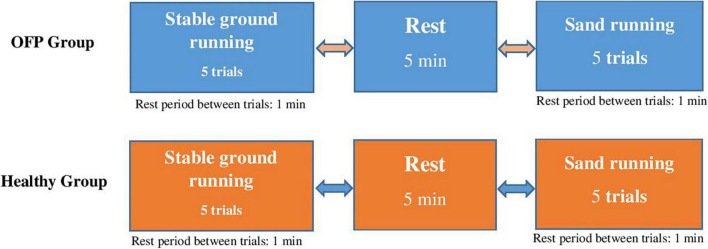
Study design with randomly applied experimental conditions. Participants ran at preferred speed. OPF stands for over-pronated feet.

The main research question of this study was to examine the effects of running on sand vs. stable ground on kinetics and muscle activities in individuals with over-pronated feet and healthy controls. To address this research question, a cross-sectional study was designed (Figure 1). Participants were tested on 1 day with sufficient rest in between the experimental conditions to minimize the effects of fatigue. The experimental conditions were sequenced in random order.

### Participants

The freeware tool G × Power (Version 3.1.9.2, University of Kiel, Germany) was used to compute a one-sided *a priori* power analysis with the *F*-test family (ANOVA for repeated measures, within-between interaction) and the respective statistical test based on a related study that examined walking on sand kinetics in adults with OPF (Jafarnezhadgero A. et al., 2019). The power analysis was calculated with an assumed Type I error of 0.05, a Type II error rate of 0.20 (actual power 0.80), and an effect size of 0.80 (i.e., interaction effects) for walking kinetics (i.e., peak vertical GRF). The analysis revealed that 20 participants per group would be sufficient to observe significant group-by-time interactions. Due to potential drop-outs, 33 healthy individuals (21 females, 12 males) aged 22.2 ± 2.5 years (body height: 178.0 ± 6.6 cm; mass: 75.0 ± 8.2 kg; body-mass-index: 23.8 ± 3.4 kg/m^2^) and 33 OPF individuals (24 females, 9 males) aged 22.2 ± 1.9 years (body height: 163.9 ± 5.5 cm; mass: 68.4 ± 18.4 kg; body-mass-index: 25.2 ± 5.3 kg/m^2^) were enrolled in this study. All participants were sedentary and were not physically active according to WHO guidelines (150 min per week of moderate to vigorous physical activity). All participants were right footed as determined by a kicking ball test. Participants were included in the OPF group, if they showed a navicular drop of>10 mm, and a foot posture index of>10 (Farahpour et al., 2018). For the control group, participants were eligible to be included if they showed a navicular drop of<10 mm and>6 mm, and a foot posture index of<5 and>0 (Redmond et al., 2006). Navicular drop (OPF: 13.1 ± 0.8 mm; control: 7.3 ± 0.6 mm) and foot posture index (OPF: 11.1 ± 0.7; control: 3.2 ± 1.1) in the OPF group were greater than that control group (*p* < 0.001). In the current study, a modified version of the navicular drop described by Brody (1982) was used to determine the sagittal plane displacement of the navicular between seated position and standing on one leg. During testing, participants were seated on a chair with both feet flat on the ground and knees flexed at an angle of 90°. The most medial aspect of the navicular was marked. The height of the navicular was measured using a ruler. Thereafter, the participant was asked to stand on one leg by flexing the contralateral knee. The single-limb stance position was used because recent work by McPoil and Cornwall (1996) has shown that measurements taken from this position more accurately represent the position of the foot during the midstance phase of walking. Again, the height of the navicular was measured using a ruler. The difference between the height of the navicular in seated position vs. standing on one leg was recorded as navicular drop. The foot posture index was evaluated by a podiatrist with ∼10 years of professional experience. For this purpose, a visual analog scale was used. The foot posture index consists of six items used to quantify and classify foot posture (Redmond et al., 2006; Gijon-Nogueron et al., 2015). These are (i) palpation of the head of the talus; (ii) curvatures above and below the lateral malleolus; (iii) position of the calcaneus in the frontal plane; (iv) prominence of the malleolus; (v) congruence of the medial longitudinal arch; and (vi) abduction/adduction of the forefoot. Each item was rated on a visual analog scale ranging from –2 to +2, resulting in a total score of –12 to +12. Negative values indicate a supinated foot posture, and positive values indicate a pronated foot posture. Of note, values of 10–12 in the foot posture index were classified as over-pronated feet (Redmond et al., 2006; Gijon-Nogueron et al., 2015). A detailed description of the foot posture index can be found elsewhere (Redmond et al., 2006; Gijon-Nogueron et al., 2015). Exclusion criteria comprised for both groups a history of musculoskeletal surgery at the lower limbs, neuromuscular or postural disorders (except for OPF individuals), and limb length differences larger than 5 mm. All participants were rearfoot strikers. The Institutional Review Boards of the Medical Sciences University of Ardabil, Iran approved the research protocol (IR.ARUMS.REC.1398.119). Prior to the start of the study, written informed consent was obtained from all participants.

### Apparatus and Data Processing

**FIGURE 2:**
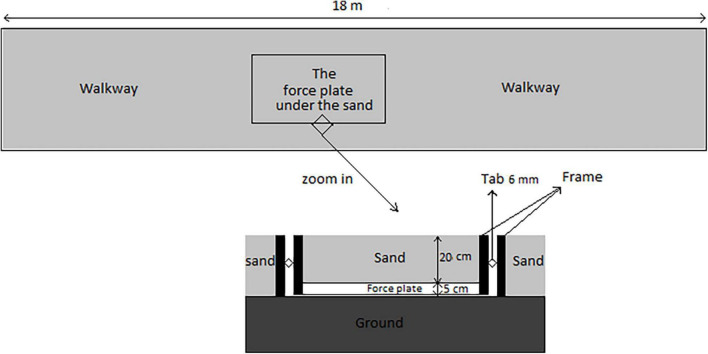
Schematic image of the runway and the location of the force plate.

The experimental study design included two runways (18-m long, 1-m wide, and 0.25-m deep) with a specifically constructed frame (Jafarnezhadgero A. et al., 2019). The stable runway was covered with reinforced plywood and an embedded force plate (Bertec Corporation, Columbus, OH, United States). The unstable runway was filled with dry sand to simulate a common deformable surface environment. A Bertec force plate with a sampling rate of 1,000 Hz was embedded in the runway (20 cm underneath the sand; Xu et al., 2015) and used to collect GRF data. The frame consisted of two steel rectangles concentrically aligned with 6 mm clearance between the walls (Figure 2). Previous studies showed that this runway construction is well-suited to accurately assess GRFs while running (Merryweather, 2008; Yoo, 2014; Xu et al., 2015). The force plate was embedded halfway through the 18 m runway. The participants started the test trial on the runway and ran a distance of 9 m before they stepped on the base plate. Running speed was monitored using two sets of infrared photocells.

Kinetic data were recorded in accordance with a previous study (Jafarnezhadgero A. et al., 2019). In brief, a 20 Hz low-pass filter (4th order Butterworth filter, zero lag) was used to process GRF data (Damavandi et al., 2012). Heel strike and toe off events during running were identified using the Bertec force plate. Accordingly, a 10 N threshold was used to detect the stance phase of the running trials. GRF data were normalized to body weight (BW). The x, y, and z force components represent medio-lateral, antero-posterior, and vertical directions. GRF data were analyzed by computing the first (Fz_*HC*_) and second vertical peak force (Fz_*PO*_) (Jafarnezhadgero et al., 2017). In addition, braking (Fy_*HC*_) and propulsion forces (Fy_*PO*_) were recorded from the anterior-posterior force curve. The positive (lateral) peak (Fx_*HC*_) and the negative (medial) peak (Fx_*PO*_) were additionally calculated (Jafarnezhadgero et al., 2017). Time-to-peak (TTP) was defined as the time between the initial heel contact and the respective Fz_*HC*_ peak value. Vertical loading rates were computed as the average slope between 20 and 80% of the vertical GRF (Jafarnezhadgero et al., 2017). For all computed running parameters, the average of three trials was used for statistical analyses.

A wireless EMG system (Biometrics Ltd., Nine Mile Point Ind. Est, Newport, United Kingdom) with eight pairs of bipolar Ag/AgCl surface electrodes was applied to assess tibialis anterior (TA), gastrocnemius medialis (Gas-M), biceps femoris (BF), semitendinosus (ST), vastus lateralis (VL), vastus medialis (VM), rectus femoris (RF), and gluteus medius (Glut-M) muscle activities of the dominant limb (Farahpour et al., 2018). Center-to-center electrode distance was 25 mm. Input impedance and common mode rejection ratio was set at 100 MΩ and>110 dB, respectively. The raw EMG signals were digitized at 1,000 Hz. According to the European recommendations for surface electromyography (SENIAM), skin surface was shaved and cleaned with alcohol (70% Ethanol–C_2_H_5_OH) over the respective muscle bellies. Thereafter, the skin was gently abraded before electrode placement. The surface electrodes were placed on the muscle belly, longitudinally to the muscle fibers (Farahpour et al., 2018). GRFs and EMG data were synchronized using Nexus software (Oxford Metrics, Oxford, United Kingdom). EMG data were processed in accordance with a previous study (Farahpour et al., 2018). In brief, test-trials were divided into the loading (0–15% of running cycle), mid-stance (15–25% of running cycle), and push-off (25–40% of running cycle) phases (Ounpuu, 1994; Dugan and Bhat, 2005). Maximum voluntary isometric contraction (MVIC) was assessed using a handheld dynamometer to normalize EMG during running to MVIC.

### Experimental Procedures

During testing, all participants were equipped with the same shoe model (Adidas, Climacool vento shoes) that best fitted the size of their foot. This shoe model was used for participants with a normal foot. The midsole drop of these shoes were 10 mm (heel: 20.5 mm/forefoot: 10.5 mm). Before the start of the study, participants were familiarized with the applied tests and instruments. Participants performed two familiarization trials before the actual tests were recorded. For each participant, 7 trials were recorded and on average, 5 trials were successful, i.e., provided useful data. During testing, individuals were kindly asked to run at preferred speed and in randomized order over stable ground and sand. A 5 min rest was scheduled between running conditions and a 1 min rest between running trials to minimize the effects of fatigue (Figure 1). A trial was considered successful if the foot landed in the middle of the force plate and if EMG signals were artifact free upon visual examination of the online screen. All participants were rearfoot strikers and the foot strike was monitored between conditions. MVIC tests were applied after the running analyses for each muscle separately to normalize EMG data.

### Statistical Analyses

The Shapiro-Wilk-Test confirmed normal data distribution. Accordingly, data were presented as group mean values and standard deviations. To examine the effect of ground surface during running, a separate 2 (group: healthy vs. OPF) × 2 (surface: stable ground vs. sand) ANOVA with repeated measures was computed on speed adjusted (i.e., differences between running on sand vs. stable ground) GRF and EMG variables. *Post-hoc* analyses were computed using Bonferroni adjusted paired sample *t*-tests. In addition, effect sizes were calculated by converting partial eta-squared (η^2^*_*p*_*) to Cohen’s d. According to Cohen, *d* < 0.50 indicate small effects, 0.50 ≤ *d* < 0.80 indicate medium effects, and *d* ≥ 0.80 indicate large effects. The significance level was set at *p* < 0.05. The Statistical Package for Social Sciences (SPSS) version 24.0 was used to calculate inferential statistics.

## Results

### Running Speed

**FIGURE 3:**
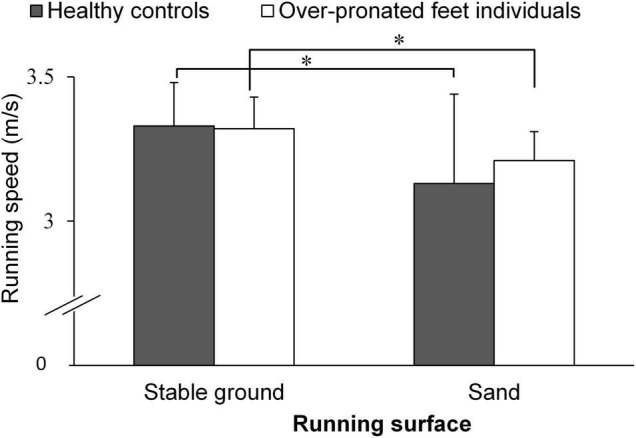
Running speed in both groups. *Indicates significant within group differences.

A significant main effect of “surface” was found for running speed (*p* < 0.001; *d* = 1.38). The *post-hoc* test demonstrated that running on sand (3.17 ± 0.23 m/s) resulted in lower speed compared with stable ground running (3.32 ± 0.13 m/s) (*p* < 0.001; *d* = 0.83; 95% CI: 0.02, 0.27). No significant main effects of “group” and group-by-surface interactions were found for running speed (*p* > 0.05; *d* = 0.23–0.39) (Figure 3).

### Running Kinetics

The speed adjusted analysis demonstrated significant main effects of “surface” for Fy_*HC*_ and Fy_*PO*_ (*p* < 0.002; *d* = 0.86–1.02) (Table 1). *Post-hoc* tests revealed significantly lower Fy_*HC*_ (*p* < 0.001; *d* = 0.88) and Fy_*PO*_ (*p* = 0.001; *d* = 0.86) when running on sand compared with stable ground. Moreover, we observed significant main effects of “group” for Fz_*PO*_ and loading rate (*p* < 0.015; *d* = 0.37–0.63). *Post-hoc* tests showed significantly lower Fz_*PO*_ (*p* = 0.014; *d* = 0.59) and larger loading rates (*p* = 0.004; *d* = 0.72) in OPF individuals compared with controls (Table 1). No statistically significant group-by-surface interactions were found for loading rate during running (*p* > 0.05; *d* = 0.00–0.24).

**TABLE 1:** Data are means and standard deviations for ground reaction forces (GRF) during running on sand vs. stable ground in over-pronated feet (OPF) individuals compared with healthy controls.

GRF	Healthy controls				OPF individuals			Δ%	Sig. (Effect size)		
	Stable ground	Sand	95% CI	Δ%	Stable ground	Sand	95% CI		Surface	Group	Group × surface
Fz_HC_ (% BW)	93.16 ± 31.71	96.51 ± 28.94	–15.66, 8.94	3.59	109.83 ± 36.11	114.18 ± 31.22	–14.20, 5.51	3.96	0.648 (0.11)	0.056 (0.49)	0.998 (0.00)
Fz_PO_ (% BW)	190.34 ± 24.48	188.41 ± 33.07	–4.48, 8.34	–1.01	175.12 ± 24.16	175.73 ± 29.17	–9.37, 8.17	0.34	0.331 (0.24)	**0.014 (0.63)**	0.840 (0.06)
Fx_HC_ (% BW)	6.36 ± 3.71	5.53 ± 3.74	–1.16, 2.81	–13.05	8.25 ± 4.91	6.45 ± 4.84	–0.40, 4.01	–21.81	0.086 (0.43)	0.090 (0.43)	0.459 (0.19)
Fx_PO_ (% BW)	–6.62 ± 3.73	–6.22 ± 6.94	–2.74, 1.95	–6.04	–5.22 ± 4.92	–6.31 ± 5.50	–1.22, 3.40	20.88	0.972 (0.00)	0.455 (0.19)	0.435 (0.20)
Fy_HC_ (% BW)	–9.90 ± 8.64	–4.08 ± 2.09	–8.64, –2.99	–58.78	–8.69 ± 8.59	–5.08 ± 2.15	–6.64, –0.56	–41.54	**<0.001 (1.02)**	0.938 (0.00)	0.339 (0.24)
Fy_PO_ (% BW)	7.38 ± 5.71	4.02 ± 1.39	1.07, 5.64	–45.52	5.91 ± 3.83	3.95 ± 1.97	0.44, 3.47	–33.16	**0.001 (0.86)**	0.222 (0.15)	0.348 (0.23)
TTP Fz_HC_ (ms)	25.48 ± 14.99	36.69 ± 57.95	–30.10, 7.68	43.99	20.75 ± 15.48	22.18 ± 17.12	–6.21, 3.36	6.89	0.277 (0.27)	0.204 (0.32)	0.327 (0.24)
Loading rate (BW/s)	47.85 ± 33.08	45.56 ± 24.11	–7.70, 12.29	–4.78	79.99 ± 56.89	71.00 ± 39.68	–9.51, 27.50	–11.23	0.976 (0.00)	**0.004 (0.37)**	0.734 (0.09)

*BW, body weight; x, medio-lateral direction; y, anterior-posterior direction; z, vertical direction; Fz_HC,_ peak vertical ground reaction force during heel contact; Fz_PO,_ peak vertical ground reaction force during the push-off phase; Fy_HC_, braking force; Fy_PO_, propulsion force; Fx_HC_, peak lateral ground reaction force during heel contact; Fx_PO_, peak medial ground reaction force during the push-off phase; TTP, time-to-peak; 95% CI, confidence interval refers to the confidence interval of the difference between stable ground and sand condition. Significant results were denoted in bold.*

### Muscle Activities

With regards to muscle activities, the speed adjusted analysis revealed statistically significant main effects of “surface” for TA and Gas-M activities during the loading phase (*p* < 0.033; *d* = 0.55–0.58) (Table 2). *Post-hoc* tests showed significantly higher TA activities (*p* = 0.024; *d* = 0.28) and lower Gas-M activities (*p* = 0.032; *d* = 0.43) when running on sand compared with stable ground.

**TABLE 2:** Data are means and standard deviations for muscle activity during the loading phase [% maximum voluntary isometric contraction (MVIC)] when running on stable ground and sand.

Muscles	Healthy controls				OPF individuals				Sig. (Effect size)		
	Stable ground	Sand	95% CI	Δ%	Stable ground	sand	95% CI	Δ%	Surface	Group	Group × surface
TA	32.68 ± 15.34	38.08 ± 17.53	–9.64, –1.15	16.52	34.56 ± 20.06	37.07 ± 27.45	–7.27, 2.25	7.26	**0.024 (0.58)**	0660 (0.11)	0.422 (0.20)
Gas-M	11.62 ± 15.14	8.99 ± 10.34	0.28, 4.96	–22.63	14.06 ± 9.66	13.98 ± 9.16	–2.37, 2.52	–0.56	**0.032 (0.55)**	0.105 (0.41)	0.224 (0.30)
VL	15.03 ± 14.21	17.92 ± 19.91	–10.78, 5.00	19.22	28.43 ± 60.72	24.19 ± 25.83	–17.51, 25.98	–14.91	0.872 (0.00)	0.051 (0.50)	0.529 (0.15)
VM	17.88 ± 12.34	21.44 ± 23.51	–12.26, 5.15	19.91	34.59 ± 28.95	40.03 ± 34.31	–18.66, 7.77	15.72	0.254 (0.29)	**<0.001 (0.93)**	0.759 (0.09)
RF	17.32 ± 7.41	17.36 ± 6.87	–2.39, 2.32	0.23	29.89 ± 20.91	29.40 ± 23.32	–4.40, 5.38	–1.63	0.469 (0.18)	**0.001 (0.91)**	0.916 (0.00)
BF	20.95 ± 20.91	21.15 ± 30.12	–6.28, 5.86	0.95	23.59 ± 16.96	22.54 ± 21.19	–6.98, 9.09	–4.45	0.337 (0.24)	0.843 (0.06)	0.594 (0.14)
ST	17.85 ± 16.37	19.37 ± 21.55	–6.67, 3.64	8.51	26.96 ± 20.12	23.74 ± 16.97	–2.37, 8.81	–11.94	0.588 (0.14)	0.132 (0.38)	0.349 (0.23)
Glut-M	32.68 ± 28.34	25.05 ± 22.95	–1.58, 16.85	–23.34	43.02 ± 34.35	37.03 ± 24.48	–3.93, 15.92	–13.92	0.213 (0.31)	0.103 (0.41)	0.701 (0.09)

*OPF, over-pronated feet; TA, tibialis anterior; Gas-M, gastrocnemius medialis; BF, biceps femoris; ST, semitendinosus; VL, vastus lateralis; VM, vastus medialis; RF, rectus femoris; Glut-M, gluteus medius; 95% CI, confidence interval refers to the confidence interval of the difference between stable ground and sand condition. Significant results were denoted in bold.*

Significant main effects of “group” were observed for VM and RF activities (*p* < 0.002; *d* = 0.91–0.93) during the loading phase (Table 2). *Post-hoc* tests showed significantly larger VM (*p* < 0.001; *d* = 0.89) and RF (*p* = 0.001; *d* = 0.61) activities during the loading phase in OPF individuals compared with healthy controls.

No statistically significant group-by-surface interactions were detected for activities of selected lower limb muscles during the loading phase (*p* > 0.05; *d* = 0.00–0.30) (Table 2).

The speed adjusted analysis showed significant main effects of “surface” for Gas-M, VL, and RF activities during the mid-stance phase (*p* < 0.033; *d* = 0.55–0.58) (Table 3). *Post-hoc* tests revealed significantly lower Gas-M (*p* = 0.005; *d* = 0.50) and VL (*p* = 0.027; *d* = 0.28) activities and larger RF activities (*p* = 0.044; *d* = 0.30) when running on sand.

**TABLE 3:** Data are means and standard deviations for muscle activity during the mid-stance phase [% maximum voluntary isometric contraction (MVIC)] when running on stable ground and sand.

Muscles	Healthy controls				OPF individuals				Sig. (Effect size)		
	Stable ground	Sand	95% CI	Δ%	Stable ground	Sand	95% CI	Δ%	Surface	Group	Group × surface
TA	22.77 ± 13.83	27.83 ± 21.62	–11.97, 1.84	22.22	30.16 ± 23.57	31.43 ± 17.62	–6.56, 4.01	4.21	0.076 (0.45)	0.159 (0.35)	0.506 (0.16)
Gas-M	57.09 ± 22.14	48.32 ± 35.90	–4.06, 21.85	–15.36	94.40 ± 49.74	78.79 ± 42.91	4.76, 26.46	–16.53	**0.024 (0.58)**	**<0.001 (1.00)**	0.441 (0.19)
VL	69.67 ± 55.11	57.67 ± 35.04	–4.09, 28.09	–17.22	60.21 ± 55.66	53.01 ± 43.60	–13.28, 27.69	–11.95	**0.026 (0.57)**	0.600 (0.12)	0.981 (0.00)
VM	71.83 ± 53.50	58.66 ± 35.84	–3.59, 29.91	–18.33	60.82 ± 46.60	64.24 ± 40.68	–23.62, 16.77	5.62	0.996 (0.00)	0.873 (0.00)	0.145 (0.36)
RF	27.33 ± 16.20	36.82 ± 20.60	–14.60, –4.37	34.72	45.54 ± 41.10	51.29 ± 42.95	–21.68, 10.18	12.62	**0.032 (0.55)**	**0.021 (0.59)**	0.825 (0.06)
BF	34.65 ± 39.40	28.65 ± 40.15	–4.38, 16.37	–17.31	24.83 ± 16.26	27.18 ± 16.84	–8.10, 3.41	9.46	0.971 (0.00)	0.380 (0.22)	0.123 (0.39)
ST	18.76 ± 23.99	17.91 ± 29.28	–6.05, 7.74	–4.53	28.60 ± 25.33	31.98 ± 32.34	–11.08, 4.32	11.81	0.758 (0.09)	0.174 (0.34)	0.440 (0.19)
Glut-M	62.80 ± 46.67	37.53 ± 36.97	10.84, 39.69	–40.23	54.45 ± 50.02	55.03 ± 41.58	–17.45, 16.29	1.06	0.427 (0.20)	0.725 (0.09)	**0.008 (0.69)**

*OPF, over-pronated feet; TA, tibialis anterior; Gas-M, gastrocnemius medialis; BF, biceps femoris; ST, semitendinosus; VL; vastus lateralis; VM, vastus medialis; RF, rectus femoris; Glut-M, gluteus medius; 95% CI, confidence interval refers to the confidence interval of the difference between stable ground and sand condition. Significant results were denoted in bold.*

Significant main effects of “group” were found for Gas-M and RF activities (*p* < 0.022; *d* = 0.59–1.00) during the mid-stance phase of running (Table 3). *Post-hoc* tests showed significantly larger Gas-M (*p* < 0.001; *d* = 0.95) and RF (*p* = 0.020; *d* = 0.65) activities in OPF individuals compared with controls.

The statistical analysis revealed significant group-by-surface interactions for Glut-M activities (*p* = 0.008; *d* = 0.69) during the mid-stance phase of running (Table 3). Control but not OPF individuals showed significantly lower Glut-M activities (*p* = 0.022; *d* = 0.63) when running on sand.

The speed adjusted analysis revealed significant main effects of “surface” for Gas-M and ST activities during the push-off phase (*p* < 0.006; *d* = 0.72–1.26) (Table 4). *Post-hoc* tests indicated lower Gas-M (*p* < 0.001; *d* = 1.05) and ST (*p* = 0.046; *d* = 0.34) activities when running on sand.

**TABLE 4:** Data are means and standard deviations for muscle activity during the push-off phase [% maximum voluntary isometric contraction (MVIC)] when running on stable ground and sand.

Muscles	Healthy controls				OPF individuals				Sig. (Effect size)		
	Stable ground	Sand	95% CI	Δ%	Stable ground	Sand	95% CI	Δ%	Surface	Group	Group × surface
TA	16.78 ± 12.97	11.55 ± 6.73	1.13, 9.32	–31.16	17.83 ± 16.53	19.58 ± 13.47	–7.93, 4.43	9.81	0.065 (0.47)	0.091 (0.43)	0.127 (0.39)
Gas-M	103.49 ± 48.07	80.67 ± 37.92	6.82, 38.81	–22.05	134.27 ± 69.68	81.22 ± 44.87	31.35, 74.76	–39.50	**<0.001 (1.26**)	0.125 (0.39)	**0.022 (0.59)**
VL	19.03 ± 24.94	14.94 ± 24.89	–9.06, 17.24	–21.49	31.91 ± 46.20	29.80 ± 42.24	–16.11, 20.33	–6.61	0.644 (0.11)	**0.017 (0.62)**	0.863 (0.00)
VM	24.02 ± 25.72	21.33 ± 30.45	–9.36, 14.75	–11.19	33.05 ± 28.00	43.55 ± 40.48	–24.79, 3.80	31.77	0.875 (0.00)	**0.004 (0.74)**	0.226 (0.30)
RF	17.21 ± 8.35	18.53 ± 10.23	–5.00, 2.38	7.66	39.36 ± 35.19	37.80 ± 30.65	–9.93, 13.04	–3.96	0.979 (0.00)	**<0.001 (1.08)**	0.654 (0.11)
BF	17.51 ± 18.32	22.53 ± 30.52	–14.28, 4.25	28.66	17.42 ± 15.47	19.08 ± 20.15	–9.10, 5.77	9.52	0.170 (0.35)	0.622 (0.12)	0.684 (0.11)
ST	14.23 ± 14.88	9.48 ± 9.97	–0.46, 9.95	–33.38	23.70 ± 22.01	19.95 ± 21.53	–5.49, 12.99	–15.82	**0.005 (0.72)**	**0.010 (0.67)**	0.756 (0.09)
Glut-M	31.55 ± 41.06	26.38 ± 34.51	–4.47, 14.82	74.42	29.86 ± 21.85	33.66 ± 30.16	–13.74, 6.13	12.72	0.801 (0.06)	0.827 (0.06)	0.214 (0.31)

*OPF, over-pronated feet; TA, tibialis anterior; Gas-M, gastrocnemius medialis; BF, biceps femoris; ST, semitendinosus; VL, vastus lateralis; VM, vastus medialis; RF, rectus femoris; Glut-M, gluteus medius; 95% CI, confidence interval refers to the confidence interval of the difference between stable ground and sand condition. Significant results were denoted in bold.*

Significant main effects of “group” were identified for VL, VM, RF, and ST activities during the push-off phase (*p* < 0.018; *d* = 0.62–1.08) (Table 4). *Post-hoc* tests showed greater VL (*p* = 0.017; *d* = 0.60), VM (*p* = 0.004; *d* = 0.69), RF (*p* < 0.001; *d* = 1.04), and ST (*p* = 0.010; *d* = 0.65) activities in OPF individuals compared with controls.

Finally, significant group-by-surface interactions were found for Gas-M activities (*p* = 0.022; *d* = 0.59) during the push-off phase (Table 4). OPF individuals showed lower Gas-M activities when running on sand (*p* < 0.001; *d* = 0.93).

## Discussion

This study examined the effects of running on sand vs. stable ground on GRFs and activities of selected lower limb muscles in OPF individuals compared with healthy age-matched controls. The main findings of this study were that running on sand resulted in lower running speed, irrespective of the experimental group. Due to this outcome, all statistical analyses were adjusted for the observed surface-related differences in running speed. OPF individuals showed larger loading rates and greater vastus medialis and rectus femoris activities during the loading phase. Irrespective of the experimental groups, running on sand produced a higher tibialis anterior activity during the loading phase, a lower VL activity during the mid-stance phase, and a lower semitendinosus activity during the push-off phase.

This study revealed lower running speed along with lower peak posterior and anterior GRF amplitudes in both experimental groups when running on sand vs. stable ground. Given that we adjusted our statistical model for surface-related differences in running speed, it seems justified to argue that these differences were surface and not speed-related. This study showed that running on sand resulted in lower peak anterior GRFs during the push-off phase which may have been caused by a lower medial gastrocnemius activity. Of note, lower peak anterior GRF amplitudes during running on sand may indicate instability of the foot and/or ankle most likely due to deficits in the mid-tarsal locking mechanism during the late stance phase of running. It can be speculated that running on sand demands a greater effort to accelerate the body’s center of mass within the sagittal plane, i.e., plane of locomotion (Damavandi et al., 2012) to achieve similar speed compared with stable ground running.

Similar loading rates were found when running on sand vs. stable ground. During the loading phase of running on sand, higher tibialis anterior and lower medial gastrocnemius activities were observed in both groups. The loading phase of running is characterized by ankle plantarflexion. During this phase, eccentric tibialis anterior actions are needed to gently lower the foot to the ground (Whittle, 2014). This study revealed a higher tibialis anterior muscle activity when running on sand compared with firm ground.

There is evidence that the relationship between medial gastrocnemius activity and the resultant plantarflexion moment is influenced by the underlying force-length relation (Arnold et al., 2013). The medial gastrocnemius muscle contributes to knee flexion and plantarflexion of the ankle joint (Li et al., 2002). A previous study demonstrated that knee flexion was greater during the loading phase when running at different velocities (8 and 11 km.h^–1^) on sand compared with firm ground (Luheff et al., 2005). Thus, the plantarflexion moment generated through medial gastrocnemius activity when running on sand could be affected by the knee angle (Yong et al., 2014). Accordingly, greater knee flexion due to shortened medial gastrocnemius length may result in lower medial gastrocnemius activity which could cause a lower ankle plantarflexion moment (Li et al., 2002). The complex relationship between muscle length and its force-generating capacity is likely the reason why medial gastrocnemius activity is lower when running on sand.

A previous study showed an association between running on stable ground and an increased risk of sustaining lower limb injuries (James et al., 1978). The magnitude and the rate of loading have been identified as risk factors for running-related injuries (Barrett et al., 1998), and changes in running style are effective in reducing vertical loading rate (Pirscoveanu et al., 2021). The active support system for the medial longitudinal arch includes appropriate tibialis anterior muscle activity (Oatis, 2009). In this study, we showed that running on sand vs. stable ground resulted in similar loading rates but higher tibialis anterior activity when running on sand. Accordingly, running on sand could be an adequate means to enhance tibialis anterior -activity during running. Higher tibialis anterior activity may restore the medial longitudinal arch in OPF individuals. However, more research is needed on the long-term effects of running on sand to better understand this issue.

Irrespective of the experimental group, we observed similar vertical impact peaks and quadriceps activities during the loading phase when running on sand and stable ground. There is evidence that running on soft or compliant surfaces reduces impact forces due to prolonged collision time (Barrett et al., 1998). Of note, a previous study showed a sharp spike in foot impact force which amounted to five times body weight when running on hard vs. soft ground (McMahon and Greene, 1979). Previously, researchers proposed that running on sand is an effective rehabilitative means to treat lower limb injuries because it produces low impact forces and high muscle activities (Barrett et al., 1998).

During mid-stance and push-off phases, medial gastrocnemius activity was lower when running on sand compared with stable ground. In this study, we found lower medial gastrocnemius activity when running on sand compared with stable ground which could be due to the fact that sand may absorb some of the impact energy during landing. Additionally, we showed larger loading rates in OPF individuals compared with healthy controls during running on sand and stable ground, as demonstrated in previous study (Jafarnezhadgero A. A. et al., 2019). Increased loading rates and impact shocks may represent biomechanical risk factors for sustaining orthopedic injuries such as knee osteoarthritis or stress fractures (Jefferson et al., 1990). Our findings revealed larger vastus medialis and rectus femoris activities during the loading phase of running in OPF individuals compared with controls. The larger vastus medialis and rectus femoris activities during the loading phase in OPF individuals can likely be interpreted as a compensatory mechanism for lower loading rates. These results are applicable to persons who run in running shoes with a rearfoot strike. Whether our findings can be translated to barefoot runners or hindfoot strike runners has to be clarified in future research. A previous study reported that running on sand produces higher energy costs compared with running on grass (Pinnington and Dawson, 2001). In addition, running on sand compared with firm ground resulted in greater hamstring, vastus lateralis, vastus medialis, and rectus femoris activities (Pinnington et al., 2005). Walking on sand has been shown to increase vertical center-of-mass displacement (Svenningsen et al., 2019), potentially increasing energy expenditure. In addition, increased energy costs when running on sand could be caused by higher lower limb muscle activities (Pinnington et al., 2005). In this study, we found higher rectus femoris activity in OPF individuals when running on sand. While this increase in muscle activity might have a positive therapeutic effect on the lower limbs of OPF individuals, it could also increase the risk of perceiving groin pain. More research is needed in this area to clarify this issue.

To the authors’ knowledge, there is no study available that examined the effects of running on sand in OPF individuals. Therefore, it is not possible to compare our results with findings from previous studies. Our results indicate reduced semitendinosus activities in both experimental groups during the push-off phase when running on sand. In the control but not the OPF group, running on sand resulted in significantly lower gluteus medius activity during the mid-stance phase. Anatomical studies on the gluteus medius suggest that this muscle plays an important role in stabilizing the hip and pelvis (Gottschalk et al., 1989). Previous studies reported associations between OPF and hip adduction during the early stance phase of walking, landing and running (Buist et al., 2010; Kagaya et al., 2015; Farahpour et al., 2018). This OPF-related mechanism affords higher hip abductor activities, mainly of the gluteus medius muscle (Farahpour et al., 2018). Of note, a study has shown OPF-related weakness of the gluteus medius muscle which functions as hip abductor (Farahpour et al., 2018). This may again increase the risk of sustaining injuries (Bellchamber and van den Bogert, 2000). During running, the gluteus medius contracts to maintain lower limb alignment from the pelvis through the femur, knee, tibia, and finally the foot (Bird et al., 2003; Semciw et al., 2013). However, running on sand did not affect gluteus medius activity in OPF individuals. There is evidence that running on sand may reduce OPF during the stance phase and might therefore lower the risk of sustaining injuries during running (Pinnington et al., 2005). Our results showed larger vastus lateralis, vastus medialis, rectus femoris, and semitendinosus activities during the push-off phase in OPF individuals compared with controls. The reason for the difference may also be due to weaker foot and leg muscles in OPF individuals compared with controls. A recent study reported that RF thickness was significantly smaller in OPF individuals compared with healthy peers (Ashnagar et al., 2019). In contrast, Ashnagar and co-workers reported that vastus lateralis and vastus medialis muscle thickness were not different in young OPF adults compared with healthy controls (Ashnagar et al., 2019).

A few methodological limitations should be discussed in the context of this study. First, a rather small study sample was recruited. However, we computed *a priori* power analysis and the outcomes support our initial cohort size. Second, we examined the acute effects of running on sand vs. stable surface. Future studies are needed to examine the long-term effects of running on sand to establish whether sand is suitable as a preventive/rehabilitative means. Third, our participants ran at preferred speed across the walkway. Surface-related differences in running speed were noted which is why the statistical ANOVA model was adjusted for running speed. Future studies should keep running speed constant when running on sand vs. stable ground to verify our findings. Fourth, perhaps a measurement of peroneus longus would have been interesting, since it affects forefoot eversion. Future studies should assess activities of peroneus longus as well.

## Conclusion

In summary, our results demonstrate that running on sand induces reduction in speed with a concomitant increase in the activity of ankle dorsiflexors. Runners presenting over-pronated feet generate greater vertical impact loading than neutral runners, while running on sand does not provide substantial reductions in this impact loading for both groups. Moreover, over-pronated feet runners demand greater quadriceps (vastus medialis and rectus femoris) activities during the loading absorption phase of running, likely to counteract instabilities related to foot misalignment.

### Practical Applications

The results of this study demonstrated that sedentary people with over-pronated feet may be exposed to greater vertical impact loading during running when compared to sedentary people with neutral feet. Moreover, running on sand contributed only marginally to reducing the instantaneous vertical loading (∼11%) of runners with neutral and over-pronated feet, while an increased muscle activation was required from knee extensors. In practice, sedentary people considering starting a running training program should be aware of their running technique and adjust the training sessions to not overload musculoskeletal structures. Moreover, running coaches working with sedentary people should also be extra-cautious with this population, to avoid early injuries that might force clients to withdraw from practice.

## Data Availability Statement

The original contributions presented in the study are included in the article/supplementary material, further inquiries can be directed to the corresponding author/s.

## Ethics Statement

The studies involving human participants were reviewed and approved by the Institutional Review Boards of the University of Mohaghegh Ardabili, Iran approved the research protocol (IR.ARUMS.REC.1398.119). The patients/participants provided their written informed consent to participate in this study.

## Author Contributions

AJ and MS: conceptualization, methodology, and supervision. NA and AF: data curation and writing-original draft preparation. AO: methodology, writing-reviewing, and editing. UG: conceptualization, methodology, writing-reviewing, and editing. All authors have read and approved the manuscript.

## Conflict of Interest

The authors declare that the research was conducted in the absence of any commercial or financial relationships that could be construed as a potential conflict of interest.

## Publisher’s Note

All claims expressed in this article are solely those of the authors and do not necessarily represent those of their affiliated organizations, or those of the publisher, the editors and the reviewers. Any product that may be evaluated in this article, or claim that may be made by its manufacturer, is not guaranteed or endorsed by the publisher.
